# Identification of Gene Expression Changes in the Aorta of ApoE Null Mice Fed a High-Fat Diet

**DOI:** 10.3390/genes8100289

**Published:** 2017-10-24

**Authors:** Dan Xi, Jinzhen Zhao, Miao Zhao, Weijun Fu, Zhigang Guo, Hui Chen

**Affiliations:** 1 Department of Critical Care Medicine, Nanfang Hospital, Southern Medical University, No. 1023, South Shatai Road, Baiyun District, Guangzhou 510515, China; wuminqunzi@163.com (D.X.); 15622123142@163.com (J.Z.); weijunfu1974@163.com (W.F.); 2 College of Veterinary Medicine, South China Agricultural University, Guangzhou 510642, China; zm1586186423@126.com; 3 Department of Cardiology, Huqiao Medical Center, Nanfang Hospital, Southern Medical University, No. 1023, South Shatai Road, Baiyun District, Guangzhou 510515, China; guozhigang126@126.com

**Keywords:** ApoE null mice, RNA-seq, atherosclerosis

## Abstract

Atherosclerosis is a chronic multifactorial inflammatory disease with high worldwide prevalence, and has become the leading cause of death. In the present study, we analyzed global gene expression changes in the aorta of Apolipoprotein E (ApoE) null mice fed a high-fat diet by using RNA-seq. We identified a total of 280 differentially expressed genes, of which 163 genes were upregulated and 117 genes were downregulated by high-fat diet compared with normal diet. Functional clustering and gene network analysis revealed that fatty acid metabolic process is crucial for atherosclerosis. By examining of the promoter regions of differentially expressed genes, we identified four causal transcription factors. Additionally, through connectivity map (CMap) analysis, multiple compounds were identified to have anti-atherosclerotic effects due to their ability to reverse gene expression during atherosclerosis. Our study provides a valuable resource for in-depth understanding of the mechanism underlying atherosclerosis.

## 1. Introduction

Atherosclerosis is a chronic multifactorial inflammatory disease with high prevalence worldwide and has become the leading cause of death [1]. The principal clinical manifestations of atherosclerosis are represented by coronary heart disease, cerebral stroke, and peripheral vascular disease. Atherosclerosis represents a heterogeneous group of pathological phenomena that include endothelium damage, inflammation, metabolic disorder, cell proliferation, foam cell formation, and soft inflamed atherosclerotic plaque rupture [2]. The molecular mechanisms underlying atherosclerosis remain unclear.

The mouse has been a powerful model in elucidating the genetic basis of atherogenesis. More than 80 genes have been confirmed to play a role in atherosclerosis by using gene-targeted mice [3]. Apolipoprotein E (ApoE) gene has a central role in lipoprotein metabolism, where it is required for the efficient clearance of chylomicrons and very-low-density lipoproteins. Notably, the ApoE null mouse was the first model showing severe and rapid development of atherosclerotic plaques without dietary intervention [4]. Using ApoE null mice fed with a high-fat diet, a previous study has determined the global gene expression profiles associated with arthrosclerosis using the microarray approach [5].

RNA-sequencing (RNA-seq), which utilizes the latest massively parallel sequencing, has provided an alternative way to obtain mRNA expression profile at unprecedented sensitivity and accuracy [6]. In contrast to the microarray, RNA-seq has several advantages. Firstly, it is an unbiased method which is not limited to detecting pre-designed sequences [6]. Secondly, it does not suffer from cross-hybridization since DNA sequences can be mapped to unique regions of the genome [7]. Lastly, it has no upper limit for quantification [8]. In the present study, we took advantage of the RNA-seq approach to investigate the global gene expression changes in the aorta of ApoE null mice fed a high-fat diet in comparison to normal diet. Our study contributes to an increase in the knowledge on molecular mechanisms underlying atherosclerosis.

## 2. Materials and Methods 

### 2.1. Sample Collection 

ApoE-/-mice on the C57BL/6 background were obtained from Vital River Laboratory Animal Technology Corporation (Beijing, China). Regular chow and high-fat diet were purchased from Guangdong Medical Lab Animal Center (Foshan, Guangdong, China). Twenty four-week-old male mice were randomly divided into two groups. The normal diet group was fed with normal chow, while the treated group was fed with a high-fat diet containing 20% fat and 2.5% cholesterol. The mice were housed in a temperature-controlled room with 12 h light-dark cycles and free access to water and food. The treatment duration was eight weeks. At the end of the experiment, the aorta was separated with general microscope from the start of the ascending aorta to common iliac artery aortic bifurcation. The adventitia was intact. We selected three aorta samples from each group for RNA-seq analysis. Another three aorta samples from each group were prepared for further verification of RNA-seq results using quantitative real-time polymerase chain reaction (qRT-PCR). All these samples were selected randomly. All animal experiments were approved by the Animal Care and Utilization Committee of the Southern Medical University. The ethical code number is SYXX(Guangdong)2015-0056.

### 2.2. RNA-Seq

Total RNA was extracted with the TRIzol reagent (invitrogen, Carlsbad, CA, USA). The RNA quality control parameters were: A260/A280 ratio > 1.8, A260/A230 ratio > 2.0 and RIN (RNA integrity number) value > 7.0. The TruSeq RNA sample preparation kit (Illumina, San Diego, CA, USA) was used to generate RNA-Seq libraries following the manufacturer’s protocol. High throughput sequencing was conducted on the Illumina HiSeq™ 2500 system. Raw data were aligned to the mouse genome (mm9) using TopHat with default options [9]. The aligned reads were then provided as an input for the quantification gene expression using Cufflinks [10]. Differentially expressed genes were chosen according to the criteria of fold change > 2 and adjusted *p*-value < 0.05.

### 2.3. Validation by Quantitative RT-PCR

The total RNA was extracted with RNAiso Plus (TaKaRa Biotechnology, Dalian, China). Reverse transcription was performed at 37 °C for 15 min followed by 98 °C for 5 min using ReverTra Ace qPCR RT Kit (Toyobo, Osaka, Japan). Quantitative PCR was performed using THUNDERBIRD SYBR qPCR Mix (Toyobo, Osaka, Japan) on the Applied Biosystems 7500 (Life Technologies, Foster City, CA, USA). The program was as follows: 95 °C for 60 s, followed by 40 cycles of 95 °C for 15 s and 60 °C for 45 s. All reactions were run in triplicate. The *β-actin* gene was amplified as a reference gene for normalization. Data were analyzed using 2^−ΔΔCt^ method. Primer sequences were listed in Table S1.

### 2.4. Gene Ontology (GO) Analysis

GO analysis was performed by using the DAVID tool [11]. The significance cutoff for false discovery rate (FDR) was set at 0.01. The R package wordcloud was used to generate word cloud for significantly enriched GO terms. The font sizes in the word cloud were proportional to ‒log10 of FDR for each enriched GO terms.

### 2.5. Gene Network Analysis

The gene network was generated by using the STRING database v10.0 [12]. The minimum combined score was set to 0.4 (the median confidence). The Cytoscape software version 3.4 (http://www.cytoscape.org) was applied for visualization and analysis of the gene network. The degree distribution was analyzed by using the Cytoscape plugin, Network Analyzer [13]. The degree threshold value for hub genes was the mean plus two standard deviations.

### 2.6. Analysis of Transcription Factor Binding Sites (TFBS)

The putative promoter regions (1 kb upstream of transcription start site) were retrieved from the UCSC Genome Browser (http://genome.ucsc.edu/). The TESS software version 6.0 [14] was used to search for matches of position-weight matrices (PWM) available in the TRANSFAC database [15]. The cutoff value for relative score was set at 0.9. Using all genes in the genome as the background, a hypergeometric test with Benjamini–Hochberg multiple test correction was conducted using in-house PERL scripts. In the end, *p*-value < 0.01 was used as the significance threshold to identify enriched transcription factors.

### 2.7. Connectivity Map (CMap) Query

The up- and downregulated genes were designated as the gene expression signature. These signature genes were submitted simultaneously for CMap query (build02; www.broadinstitute.org/cmap/). The enrichment score was calculated for each compound using the gene set enrichment analysis (GSEA) algorithm [16].

## 3. Results

### 3.1. Identification of Gene Expression Changes in the Aorta of ApoE Null Mice Fed a High-Fat Diet

**Figure 1:**
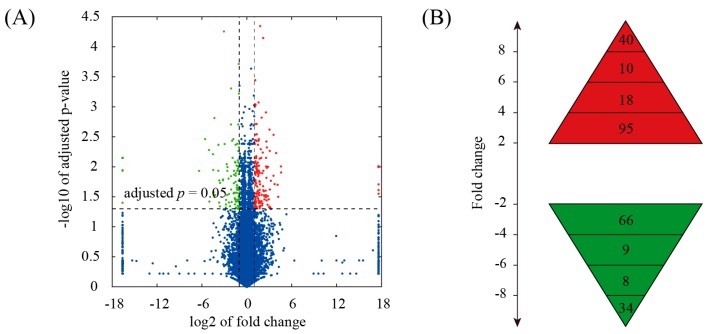

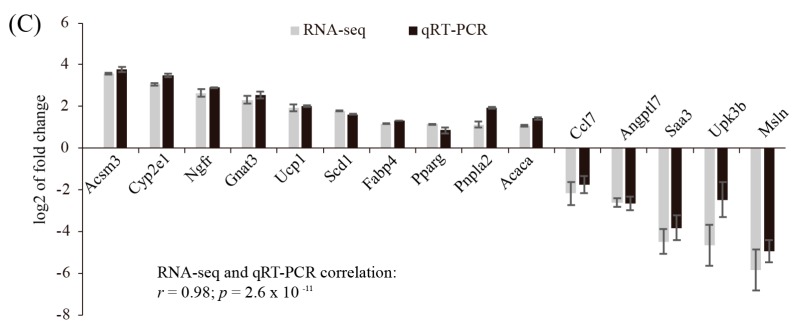
Identification of gene expression changes in the aorta of ApoE null mice fed a high-fat diet. (**A**) Volcano plot for the comparison between aorta samples from normal diet and high-fat diet fed ApoE null mice. The cutoff values for fold change and adjusted *p*-value were 2 and 0.05, respectively. Non-changed genes were shown in blue color. Red color is indicative of upregulated genes and green is indicative of downregulated genes; (**B**) Distribution of fold change values among differentially expressed genes; (**C**) Validation of selected genes identified by RNA-Seq using qRT-PCR. Fold-change values determined by both RNA-seq and qRT-PCR were presented as the mean ± SD (standard deviation).

In this study, global gene expression profile of the aorta samples from ApoE null mice fed a high-fat diet was determined by RNA-seq. The aorta samples from ApoE null mice fed a normal chow diet served as control. We obtained a total of 20.8 million reads (single-end; 50 bp in length), 81.3% of which were uniquely mapped to the mouse genome. Mapped reads were used to estimate normalized transcription level as fragments per kilobase of transcript per million mapped fragments (FPKM). Using a fold change cutoff of 2 and an adjusted *p*-value cutoff of 0.05, we identified a total of 280 differentially expressed genes (Figure 1A). Among them, 163 genes were upregulated and 117 genes were downregulated in high-fat diet group compared to normal diet group (Table S2). The range of fold changes was shown in Figure 1B. To validate the RNA-seq data, a total of 16 genes with various fold changes were selected and quantified by quantitative RT-PCR (qRT-PCR). Although there was variation in fold changes between qRT-qPCR and RNA-seq, the expression patterns were coincident between these two techniques (Figure 1C).

### 3.2. Characterizing Differentially Expressed Genes by Gene Ontology and Pathway Analysis

**Figure 2:**
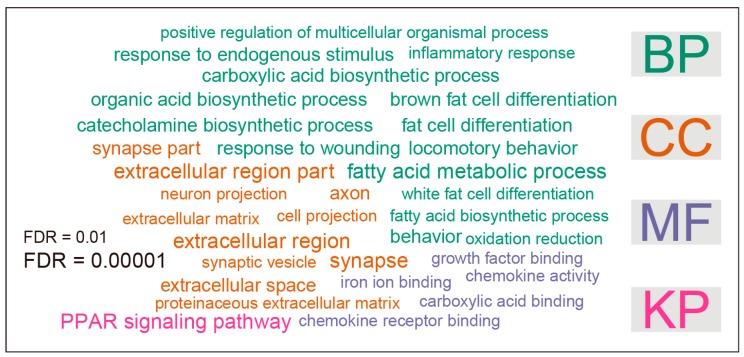
Gene ontology (GO) and pathway analysis of differentially expressed genes. The enrichment test was performed by using the DAVID tool. The significance cutoff for false discovery rate (FDR) was set at 0.01. The font sizes in the word cloud were proportional to ‒log10 of FDR for each enriched GO terms. GO terms were arranged in three categories: biological process (BP), cellular component (CC), and molecular function (MF), respectively. Pathway analysis was based on KEGG pathway (KP) annotations.

Gene ontology (GO) analysis was performed to infer the functional consequences of differentially expressed genes. Over-represented GO terms, grouped in the three categories—biological process (BP), cellular component (CC) and molecular function (MF)—were identified by using the DAVID tool. The significance cutoff for FDR was set at 0.01. There were a total of 31 over-represented terms (Figure 2). In the BP category, 15 terms were significantly enriched, including fatty acid metabolic process, behavior, locomotor behavior, brown fat cell differentiation, response to wounding, catecholamine biosynthetic process, fat cell differentiation, organic acid biosynthetic process, carboxylic acid biosynthetic process, response to endogenous stimulus, fatty acid biosynthetic process, oxidation reduction, white fat cell differentiation, inflammatory response, and positive regulation of multicellular organismal process. The 11 over-represented GO terms under the CC category were extracellular region part, extracellular region, synapse, synapse part, axon, extracellular space, extracellular matrix, proteinaceous extracellular matrix, cell projection, neuron projection, and synaptic vesicle. With respect to the MF category, five terms were significantly enriched, including iron ion binding, chemokine activity, carboxylic acid binding, chemokine receptor binding, and growth factor binding. In addition to GO analysis, we also performed pathway analysis by using DAVID online tools. In the present study, differentially expressed genes were mapped to Kyoto Encyclopedia of Genes and Genomes (KEGG) pathways. Only one pathway, namely peroxisome proliferator-activated receptors (PPAR) signaling pathway, was significantly enriched (Figure 2).

### 3.3. Searching for Hub Genes by Gene Network Analysis

**Figure 3:**
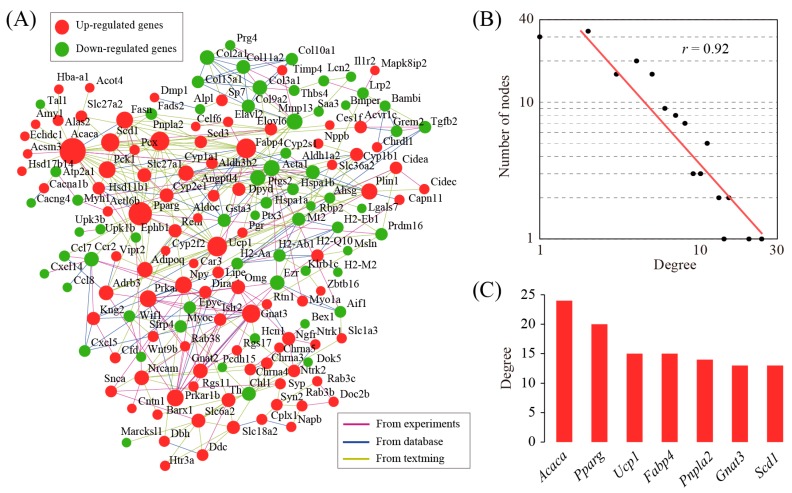
Gene network analysis of differentially expressed genes. (**A**) The structure of gene network underlying differentially expressed genes. This analysis was performed by using the STRING software. Nodes represent genes and edges represent gene–gene interactions. The diameter of each node is proportional to its degree value. This graph was generated using the Cytoscape software; (**B**) The degree distribution of the network, which follows a power law distribution; (**C**) Identification of hub genes in the gene network.

To develop a thorough picture of differentially expressed genes at the systems level, gene network analysis was performed by using integrative gene–gene interaction data from the STRING database. The reconstructed gene network consisted of 159 genes and 348 gene–gene interactions (Figure 3A). Degree distribution analysis showed that the network followed a power-law distribution (Figure 3B) and therefore belonged to scale-free small world networks [17]. Small world networks have the particular feature that some nodes, known as hub genes, are highly connected compared with others. Using a defined threshold value, we identified seven hub genes (Figure 3C). These hub genes represent functionally important genes due to their key positions in the network and thus deserve further investigation.

### 3.4. Regulatory Mechanisms Revealed by the Analysis of Transcription Factor Binding Sites

**Figure 4:**
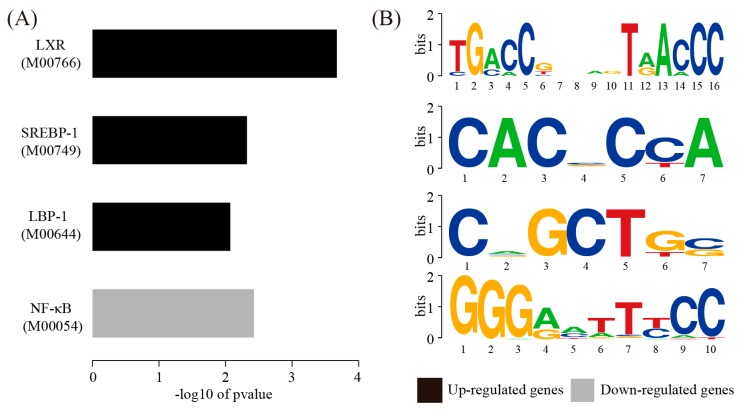
Analysis of transcription factor binding sites. (**A**) Transcription factor analysis performed on the upregulated genes and downregulated genes, respectively; (**B**) The sequence logo for enriched transcription factors.

Transcription factors are key regulator of gene expression. Up- and downregulated genes were separately tested for over-representation of transcription factor binding sites. For upregulated genes, the binding sites of LXR (M00766), SREBP-1 (M00749), and LBP-1 (M00644) were significantly over-represented. For downregulated genes, only the NF-κB (M00052) binding sites were significantly over-represented (Figure 4). This analysis provides clues to the regulatory mechanisms underlying atherosclerosis.

### 3.5. Drug Repositioning via Connectivity Map Analysis of Differentially Expressed Genes

**Figure 5:**
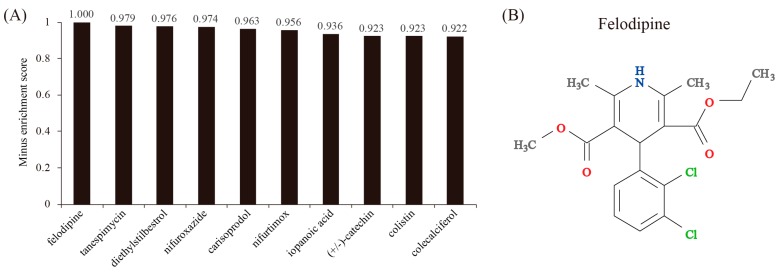
Connectivity map (CMap) analysis. (**A**) The enrichment scores of the top 10 chemical drugs resulting from CMap analysis. Differentially expressed genes were queried into CMap and chemical drugs showing a negative enrichment score were considered. (**B**) The molecular structure of the top-ranked chemical drug, felodipine.

The upregulated and downregulated genes were designated as a gene expression signature for atherosclerosis. To identify compounds that may exert anti-atherosclerotic effects due to their ability to reverse gene expression during atherosclerosis, we performed a CMap analysis in which we searched for drugs that have a gene expression pattern negatively correlating to our gene expression signature for atherosclerosis. Figure 5A showed the top 10 most promising repositioned compounds/drugs generated via CMap analysis. Among them, felodipine ranked the first (Figure 5B). There are a total of 37 genes whose regulation could be potentially reversed by felodipine, of which 24 upregulated genes are repressed (Table S3) and 13 downregulated genes are induced by felodipine (Table S4). The idea that felodipine can be repurposed to treat atherosclerosis deserves further investigation.

## 4. Discussion

In the present study, we investigated the genome-wide gene expression profiles in the aorta of ApoE null mice fed a high-fat diet compared with a normal diet using RNA-seq. A total of 280 genes, including 163 upregulated and 117 downregulated genes, were identified to be differentially expressed between the two groups. Quantitative RT-PCR (qRT-PCR) analysis demonstrated that the expression trend of selected genes was consistent with RNA-seq, suggesting that our data were of high quality.

In order to explore the functions of these differentially expressed genes, we conducted gene ontology (GO) and pathway analysis. As expected, we found fatty acid metabolic process was the most enriched term under the biological process (BP) category of GO. Notably, inflammatory response was also enriched, although not among the top-ranked GO terms. In addition, we found that PPAR signaling pathway was the most enriched pathway in KEGG pathway analysis. PPAR signaling pathway plays a central role in fatty acid metabolic process and atherosclerosis [18]. Network analysis was performed to identify seven hub genes: acetyl-coenzyme A carboxylase alpha (*Acaca*), peroxisome proliferator activated receptor gamma (*Pparg*), uncoupling protein 1 (*Ucp1*), fatty acid binding protein 4 (*Fabp4*), patatin-like phospholipase domain containing 2 (*Pnpla2*), guanine nucleotide binding protein alpha transducing 3 (*Gnat3*), and stearoyl-coenzyme A desaturase 1 (*Scd1*). Acaca is an important enzyme involved in the synthesis of saturated fatty acids, which is a known risk factor for cardiovascular diseases. Pparg plays a crucial role in the expression of key genes involved in adipogenesis, and lipid and glucid metabolism. Conditional knockout of macrophage Pparg increases atherosclerosis in C57BL/6 and low-density lipoprotein receptor-deficient mice [19]. Ucp1 is a key mitochondrial protein involved in thermogenesis in brown adipose tissue. Ucp1 over-expression in aortic smooth muscle cells causes hypertension and increases dietary atherosclerosis without affecting cholesterol levels [20]. Fabp4 belongs to a family of intracellular lipid chaperones that is expressed in active lipid metabolic tissues. The impact of Fabp4 on atherosclerosis is mainly due to the role of this molecule in macrophages and dendritic cells [21]. The pharmacological inhibition of Fabp4 significantly protected against atherosclerotic plaque formation in the ApoE-deficient animal model of atherosclerosis [22]. The *Pnpla2* gene encodes an enzyme called adipose triglyceride lipase (ATGL), which plays a role in breaking down triglycerides. Deficiency of ATGL in macrophages resulted in reduced atherosclerosis susceptibility [23]. Gnat3 plays a prominent role in taste transduction. The role of Gnat3 in atherosclerosis is unknown. Scd1 is an endoplasmic reticulum enzyme that catalyzes the rate-limiting step in the formation of monounsaturated fatty acids. Scd1 inhibition reduces atherosclerosis in cholesterol-fed C57BL/6J mice exposed to chronic intermittent hypoxia (CIH) [24]. These hub genes are likely more important than other genes due to their key positions in the network. According to GO annotations, all these hub genes except *Gnat3*, are involved in fatty acid metabolic process. Thus, the network analysis once again highlights the role of fatty acid metabolic process in atherosclerosis.

Furthermore, we predicted the transcription factors which might drive the expression of differentially expressed genes by enrichment test. We found that the binding sites of LXR (M00766), SREBP-1 (M00749), and LBP-1 (M00644) were significantly over-represented among upregulated genes. LXR (liver X receptor) is known to modulate cholesterol and fatty acid homeostasis as well as inflammation in macrophages in the context of atherosclerosis [25]. SREBP-1 (sterol regulatory element-binding transcription factor 1) belongs to a family of basic helix-loop-helix-leucine zipper (bHLHLZ) transcription factors [26]. Known target genes of SREBP-1 are involved in cholesterol biosynthesis and transport [27,28]. Interestingly, two hub genes for differentially expressed gene network, *Ucp1* and *Pnpla2*, are predicted target genes for SREBP-1 in our transcription factor analysis. This result indicates that SREBP-1 may be a crucial regulator for differentially expressed genes. LBP-1 (upstream binding protein 1) is a member of the NTF (neurogenic element binding transcription factor) family of transcription factors. Currently, little is known about the role of LBP-1 in regulating atherosclerosis. Only the NF-κB (M00052) binding sites were significantly over-represented among downregulated genes. NF-κB (nuclear factor kappa-light-chain-enhancer of activated B cells) is a protein complex that controls immune response, cytokine production, and cell survival [29]. Previous studies have shown that NF-κB inhibition reduces foam cell formation and atherosclerotic plaque accumulation [30,31]. Although nearly equal number of target genes can be activated and repressed by NF-κB respectively [32], the studies on repressed target genes of NF-κB are sparse. Hence, these four transcription factors deserve further investigation.

Using the CMap, we were able to identify compounds with a negatively correlating gene expression profile to that of differentially expressed genes. The top 10 most promising repositioned compounds were: felodipine, tanespimycin, diethylstilbestrol, nifuroxazide, carisoprodol, nifurtimox, iopanoic acid, (+/‒)-catechin, colistin, and colecalciferol. Felodipine is a calcium channel blocker which is used to treat high blood pressure. We predicted that felodipine is able to reverse the expression of 24 upregulated genes and 13 downregulated genes. The top-ranked upregulated gene is Acaca. Acaca has been identified as a hub gene in our network analysis. Felodipine represses the expression of Acaca, raising the possibility that the anti-atherosclerotic role of felodipine may be exerted by reducing saturated fatty acid synthesis via Acaca. Thus, felodipine deserves further investigation. By acting as a potent HSP90 inhibitor, tanespimycin is an antibiotic being studied in the treatment of cancers. Diethylstilbestrol is a synthetic form of the female hormone estrogen. Nifuroxazide is a nitrofuran antibiotic and is used to treat colitis and diarrhea. Carisoprodol is a muscle relaxant. It may work by altering communication among nerves in parts of the brain that control the sensation of pain and in the spinal cord. Nifurtimox is a nitrofuran derivative with antiprotozoal and potential antineoplastic activities. Iopanoic acid is a potent inhibitor of thyroid hormone release from thyroid gland, as well as of peripheral conversion of thyroxine (T4) to triiodothyronine (T3). (+/‒)-catechin is a flavan-3-ol, a type of natural phenol and antioxidant. Colistin is an antibiotic produced by certain strains of the bacteria *Paenibacillus polymyxa*, which is effective against most Gram-negative bacilli. Cholecalciferol, also known as vitamin D3, is a type of vitamin D found in food and used as a dietary supplement. These results demonstrate the validity of our CMap analysis, because estrogen and vitamin D are well-known to have beneficial effects on heart and vessels [33,34].

Finally, our study has some limitations. One clear limitation is the complex cell-type composition of the aorta samples. There are many different cell types including endothelial cells, smooth muscle cells, and macrophages. It is undoubtedly that application of laser-capture microdissection (LCM) to dissect the atherosclerotic plaque will provide a better resolution of the atherosclerotic transcriptome. Recently, considerable progress in RNA-seq technologies makes it possible to study the transcriptome of a single cell [35]. However, like LCM, the compromised sensitivity is the limiting factor for this approach at the moment [36]. Another limitation of the study is that the whole aorta was used for RNA-seq. Atherosclerotic lesions do not occur at random sites [37]. For example, the inner curvature of the aortic archa is an athero-susceptible site, whereas the descending thoracic aorta is an athero-protected region. This difference should be taken into consideration in further studies.

In conclusion, in the present study, using RNA-seq, we analyzed the transcriptomic differences in the aorta of ApoE null mice fed a high-fat diet compared with a normal diet. Our study provides a valuable resource for in-depth understanding of the molecular mechanisms underlying atherosclerosis.

## Supplementary Materials

The following are available online at www.mdpi.com/2073-4425/8/10/289/s1. Table S1: Primers used in this study for quantitative RT-PCR analysis, Table S2: The complete gene list of differentially expressed genes (fold change > 2 and adjusted *p*-value < 0.05). Table S3: Up-regulated genes in AS with the potential to be repressed by filodipine revealed by CMap analysis. Table S4: Down-regulated genes in AS with the potential to be induced by filodipine revealed by CMap analysis.
